# A QoE-Aware Energy Supply Scheme over a FiWi Access Network in the 5G Era

**DOI:** 10.3390/s20133794

**Published:** 2020-07-07

**Authors:** Chao He, Ruyan Wang

**Affiliations:** 1 School of Communication and Information Engineering, Chongqing University of Posts and Telecommunications, Chongqing 400065, China; d170101004@stu.cqupt.edu.cn; 2 Chongqing Key Laboratory of Optical Communication and Networks, Chongqing 400065, China; 3 Chongqing Key Laboratory of Ubiquitous Sensing and Networking, Chongqing 400065, China

**Keywords:** FiWi access network, energy efficiency, power over fiber, TWDM-PON, delay analysis

## Abstract

Integrated fiber-wireless (FiWi) should be regarded as a promising access network architecture in future 5G networks, and beyond; this due to its seamless combination of flexibility, ubiquity, mobility of the wireless mesh network (WMN) frontend with a large capacity, high bandwidth, strong robustness in time, and a wavelength-division multiplexed passive optical network (TWDM-PON) backhaul. However, the key issue in both traditional human-to-human (H2H) traffic and emerging Tactile Internet is the energy conservation network operation. Therefore, a power-saving method should be instrumental in the wireless retransmission-enabled architecture design. Toward this end, this paper firstly proposes a novel energy-supply paradigm of the FiWi converged network infrastructure, i.e., the emerging power over fiber (PoF) technology instead of an external power supply. Then, the existing time-division multiplexing access (TDMA) scheme and PoF technology are leveraged to carry out joint dynamic bandwidth allocation (DBA) and provide enough power for the sleep schedule in each integrated optical network unit mesh portal point (ONU-MPP) branch. Additionally, the correlation between the transmitted optical power of the optical line terminal (OLT) and the quality of experience (QoE) guarantee caused by multiple hops in the wireless frontend is taken into consideration in detail. The research results prove that the envisioned paradigm can significantly reduce the energy consumption of the whole FiWi system while satisfying the average delay constraints, thus providing enough survivability for multimode optical fiber.

## 1. Introduction

Ever-increasing green communication may still become one of the major concentrations for information and communication technology (ICT) development and evolution [1,2]. Meanwhile, the access networks of the ICT sector consume the greatest part of the whole communication architecture because of increasing network device and traffic volumes. International Mobile Telecommunications-2020 (IMT-2020) firstly proposed a 5G flower consisting of nine key performance indicators [3]. Importantly, except for cost-effectiveness, the other eight key performance indicators accepted by the International Telecommunication Union Radio Communications Department (ITU-R) are the user experience rate, spectral efficiency, mobility, delay, connection density, network energy efficiency, traffic density, and peak data rate. Thereinto, network energy efficiency can be further extended to the three typical application scenarios [1,2,3].

When it comes to the 5G-XHaul network architecture, the seamless convergence of the fiber-wireless (FiWi) broadband access network is a cutting-edge candidate paradigm, which provided network operators with the integrated advantages of a high reliability, large capacity, and low loss of the optical backhaul subnetwork with ubiquity, flexibility, and mobility of the wireless/cellular frontend subnetwork, as well as further provided both fixed subscribers and mobile users with an extensive broadband data transmission service [4,5,6]. Just for the fronthaul systems of the 5G and beyond networks, the seamless combination of a wireless access signal in the mm Wave bands and fiber-optic is proposed due to strong demand for a lower latency, high data rate, and ultra-density. The high performance of the radio access signal and optical fronthaul system is evaluated by optical self-heterodyne and subcarrier multiplexing intermediate frequency, respectively [7]. To compensate for the path loss in the mmWave frequency bands, except for beamforming and massive multiple-input multiple-output (MIMO), a self-steering phased array beamformer for the FiWi mobile fronthaul was first demonstrated without any external tuning management [8]. Van et al. evaluated the possibilities, challenges, and guidelines of power-saving mechanisms (PSM) for the Internet of Thing (IoT) application over a FiWi access network through adapting the optical network unit (ONU) sleep mode and discontinuous reception (DRX) mechanism for IoT devices [9]. Furthermore, multiple time-driven smart grid sensors report their monitored data to the energy control system utilizing the time-division multiplexing access (TDMA) mechanism. In addition, the enhanced smart grid based on a dependable FiWi access network can be deployed to further shed some light on big data acquisition, smart metering based on multistage stochastic programming, probabilistic availability quantification, as well as the total cost of ownership (TCO) and risk [10]. The most critical issues for FiWi networks, e.g., propagation delay diversity, quality of service (QoS) provisioning, survivability and reliability, prediction and estimation in the propagation delays, dynamic bandwidth allocation (DBA), network architecture optimization, as well as scheduling strategies, have received a great deal of attention, especially power-saving methods [11]. It is worth mentioning that FiWi access networks have been recently receiving significant attention in IoTs [9], smart grids [10], smart cities [12], as well as Tactile Internet [13].

Generally, the energy efficiency issue is one of the major challenges for an integrated FiWi access network, which can allow operators to decrease their OPEX and extend the battery life of mobile devices when considering the QoS constraint. A promising solution is converting the network components with a lower load and leisure into the sleep state, which focuses either on wireless/cellular fronthaul or optical access network (OAN) backhaul [9,14,15,16]. More specifically, for the wireless/cellular fronthaul segment, the wireless station (STA) adapts PSM, and the mesh points (MPs) are transformed into a doze in the IEEE 802.11 family in terms of WiFi coverage. Besides, user equipment (UE) makes the utmost of the timeout-driven DRX/discontinuous transmission (DTX) mechanisms in 4G-and-beyond-enabled cellular coverage. Meanwhile, for the fiber backhaul segment, either ONU sleep or the combination of optical line terminal (OLT) sleep with ONU sleep is separately leveraged in time-division multiplexed passive optical networks (TDM-PONs) and time and wavelength division multiplexed PONs (TWDM-PONs). It is important to note, however, that the existing literature that particularly concentrates on QoE-aware energy schemes over FiWi access networks covers the following several segments: (1) network topology design, by optimizing the number of network device deployment and periodically implementing network topology reconfiguration [14]; (2) energy-efficient DBA, by integrating the power scheduling of wireless frontend and fiber backhaul [15,17,18]; (3) dynamic adaptive mechanism, by adapting the power state of ONU according to the dynamic traffic profile [16,19,20]; (4) BSs energy consumption minimization with UE connection constraints, at the expense of the successful connection number of end equipment [21,22]; and (5) service class resource management-based PSM, worldwide interoperability for microwave access (WiMAX) and long-term evolution-advanced (LTE-A) possessing five and eight service classes, respectively, and PON consisting of three service classes via incorporating service class differentiation into the PSM of green wireless optical broadband access networks (WOBAN) [23].

Recently, in the power-efficient cloud radio access network (CRAN) operation, given the fact that the ONUs’ sleep phase in the envisioned remote radio heads (RRHs) was fragmented into several timeslots, the electrical power spent by both the ONU sleep and active states was only supplied by the OLT through the power over fiber (PoF) technology, so that mobile network operators can allow ONUs to efficiently operate without external power supplement [24,25,26]. However, the vision of providing external power to all ONU-MPPS will involve high costs, especially if the ONU-MPP over FiWi architecture is deployed where external power is not available, such as in remote mountainous areas, mobile communications in the military, etc. For this reason, PON utilizing PoF is considered as a key power supply technology. The combination of OLT sleep with ONU sleep was explained in the proposed FiWi network; however, the network equipment responsible for the wireless frontend and optical backhaul was not involved in the PoF-empowered power saving. In this paper, we take into consideration not only the converged ONU mesh portal points (ONU-MPPs) sleep scheduling scheme in the polling cycle time, but also the optical subscriber unit (OSU) for the PoF technology and data communication between the OLT and integrated ONU-MPPs over dedicated wavelengths.

More specifically, the main contributions and highlights of this paper are organized as follows:

- We propose a FiWi access network architecture based on a seamless integration paradigm of a multi-hop wireless mesh network (WMN) frontend and TWDM-PON backhaul. Then, to ensure the quality of service (QoS) characteristics and PSM formulation, we leverage the future-proof time-division multiplexed access (TDMA) scheme to jointly synchronize the DBA process. Meanwhile, we analyze the energy harvesting and conversion in the ONU module in detail.
- We apply the service-oriented DBA scheme to quantify timeslot allocation occupied by the active and sleep state of the ONU-MPP. In PoF-enabled energy supply, we investigate the energy-aware transmitted optical power scheme to achieve the minimum energy consumption, which can be adjusted to the optimum level.
- Given the minimized energy consumption and acceptable data communication delay, we derivate the mean tolerant end-to-end traffic delay over the envisioned FiWi access network, and the bridge of the correlation function between the QoE value and transmitted optical power.

The logical architecture of this paper is organized as follows. Section 2 reviews the related work of the traditional energy conservation for the FiWi access network. The provision of communication services to all integrated ONU-MPPs via PoF technology, instead of an external power supply, over the FiWi access network is envisioned in Section 3 from the viewpoint of network architecture. Section 4 elaborates on the mathematical model of energy consumption minimization, including joint bandwidth allocation and PSM problem formulation, PoF-enabled harvested power, and the correlation between multi-hops and QoE value. Under the human-to-human (H2H) traffic delay constraint, numerical analysis and discussions are evaluated in Section 5. Finally, Section 6 provides the conclusions.

## 2. Related Work of Energy Conservation over FiWi Network

The growing traffic volume requested by the end-users renders the energy consumption increase of the access network. Either the low-loaded or the leisure ONU-MPPs in the backhaul OAN was transformed into the sleep state as much as possible so that the hosting residual traffic can be rerouted to the other active ONU-MPPs to further improve the energy efficiency (EE) and network resource utilization [16,20]. However, the energy consumption of the smart FiWi access networks can be susceptible to the length of the ONU-MPP sleep state period in the polling cycle time. Togashi et al. demonstrated that, the longer the ONU sleep phase, the longer the traffic delay and the higher energy consumption [27]. Toward this end, minimizing the energy consumption of the FiWi candidate was created with only ONU-MPP sleep in mind, and the network components of the wireless frontend were neglected.

It is challenging to ensure that joint wireless (e.g., multi MP radio interfaces) and optical (e.g., ONU) power state scheduling is leveraged to carry out the energy-saving design. The optimal sleep state scheduling was achieved by leveraging the optical and radio frequency paradigm to reduce delays and energy consumption, and a two-step QoS-aware energy-efficient FiWi scenario serving as a benchmark was proposed [19]. A combination of the ONU sleep method with the radios off has been proposed to facilitate energy conservation, QoS provisioning, and efficient traffic rerouting, employing bonding wireless and optical power-state scheduling [28]. If the power-saving state scheduling in the two subnetworks are not synchronized with each other, all the FiWi networks would cause more energy consumption and additional delays. To address this issue, a cooperative ONU sleep mechanism was proposed to reduce the power consumption by dynamically integrating ONU sleep with the STA PSM control mechanism [29]. A comprehensive power-saving model of STA, MP, and ONU was proposed to not only synchronize the energy conservation scheduling strategy of the two subnetworks by leveraging the TDMA technology, but also to mitigate the end-to-end delay by taking advantage of an M/G/1 queuing model [16]. Importantly, both the QoS guaranteeing or energy-saving mode was mismatched in both segments, and the traffic transmission delay was increasing. The energy-saving modes in both coverage-centric WLAN and capacity-centric PON were referred to as STA PSM and ONU sleep, while the QoS policies were hybrid coordination function-controlled channel access (HCCA) and DBA, respectively. The smart integration of ONU sleep with STA PSM and DBA with HCCA was proposed to decrease the transmission delay [30].

It is worth mentioning that the Tactile Internet has recently attracted extensive research efforts due to common features of very little delays, an ultra-high reliability, H2H/machine-to-machine (M2M) coexistence, data-centric technology, as well as security. Van et al. has envisioned an M2M communication architecture over the emerging FiWi enhanced LTE networks, in which the M2M devices’ DRX mechanism in the cellular frontend and ONUs’ power-saving mode in the optical backhaul were devised to enhance energy saving and decrease packet delay comprehensively; accordingly, the semi-Markov process and M/G/1 queue paradigm were modeled analytically, respectively [13].

The first three sections take into consideration energy consumption minimization from the perspective of the IoT or H2H traffic over the FiWi access network. Conversely, when it comes to the FiWi-enhanced LTE-A HetNets, to achieve power consumption minimization, BSs should be switched into the sleep state as many times as possible. On the other hand, to acquire stable service maximization, BSs should be switched into the active state as many times as possible and consume more energy inevitably. There must be an optimal number of active BSs between power consumption minimization and stable service maximization. Toward this end, the power consumption minimization problem under stable service constraints was studied over the proposed architecture through a heuristic greedy algorithm, brute force algorithm, and snowball rolling algorithm [21,22].

On account of achieving energy saving, existing approaches have started to be implemented in the deployment of network devices, front-end backhaul cooperative energy-saving, ONU power state scheduling, reduction user access ratio, accessibility, traffic distribution, and service class differences. Nevertheless, research attention in the area of PoF technology over the promising FiWi access network is still in its infancy, in particular when considering energy consumption minimization.

## 3. PoF-Enabled Energy Supply Paradigm over FiWi Access Network

**Figure 1:**
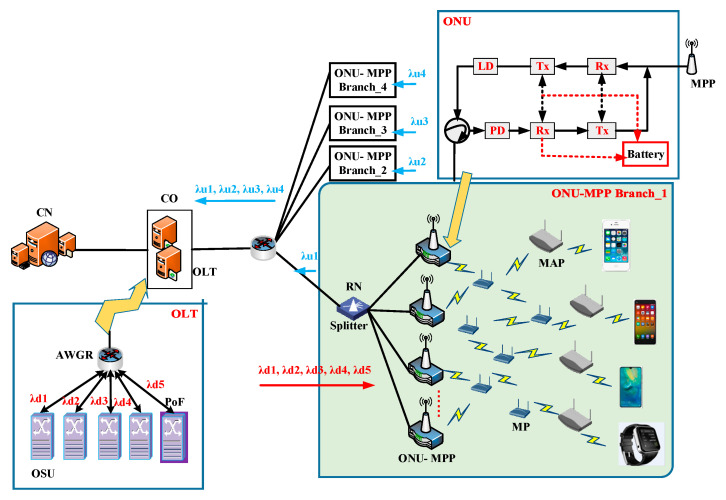
FiWi architecture illustration of the time and wavelength division multiplexed passive optical network (TWDM-PON) backhaul exploiting the power over fiber (PoF) and wireless mesh network (WMN) frontend.

The envisioned network architecture as shown in Figure 1 covers the core network (CN), bearer network, and access network, which is characterized by FiWi broadband access networks integrating a WMN frontend subnetwork adapting multi-hop with a TWDM-PON backhaul subnetwork exploiting PoF technology, as well as an optical backbone network crossing between the CN and central office (CO). Considering the PoF technology in the FiWi access network, a passive splitter in the RN is applied to provide multiple ONU-MPPs with a communication service via a single optical fiber cable. Meanwhile, an OLT associated with multiple ONU-MPPs supplies energy to each ONU-MPP with the help of the optical-fiber cable [28,31]. On the wireless network frontend, which consists of wireless access and a wireless backhaul, various kinds of new emerging terminal devices, e.g., smartphones, wearing devices, wireless sensors, unmanned aerial vehicles, connected vehicles, power grid stations, and so forth, access the integrated ONU-MPPs through an uplink PS-poll frame and downlink Beacon frame, and the ONU-MPP further allocates the requested upstream (US) subslot to each STA in the next polling cycle time by employing TDMA fashion in each ONU-MPP branch. More specially, a subset of MPs close to the terminal devices are viewed as mesh access points (MAPs), which provide the associated STAs with an access service. A subset of the MPs adjacent to the ONUs are identified as MPPs, which allocate the required bandwidth to its accommodated MPs and portion transmission subslot. Besides, other residual MPs act as relay nodes and forward the data traffic between the MAPs and ONU-MPPs. It is foreseen that the mobile access frontend in terms of the WMN paradigm provides ubiquitous connectivity for numerous smart devices and enhances access capacity.

Because of the subset of integrated ONU-MPPs in the envisioned FiWi broadband access network, consisting of an ONU module, the battery-powered module in a continuous active state, and the MPP module, which is an important network component, it exchanges data communication services between the WMN frontend and TWDM-PON optical fiber backhaul. In other words, the ONU-MPP receives the *PS-poll frames* from all the STAs and transmit the *Report frames* to the OLT in the uplink direction, while receiving the *Gate frames* from the OLT and then transmitting the *Beacon frames* to each STA in the downlink direction. In addition, the converged ONU-MPPs can flexibly switch between the sleep phase and active phase according to the service-oriented associated STAs. 

On the optical backhaul segment, the OLTs located in the CO manage the resource allocation of the integrated ONU-MPPs via wavelength-division multiplexing (WDM); broadcast the data communication traffic stemming from the CO from the OLT to multiple integrated ONU-MPPs; and provide integrated ONU-MPPs with power supply via PoF technology, which is capable of converting an optical signal, e.g., receiving unnecessary data that is not meant for the accommodated ONU-MPPs, into electrical power consumed by the integrated ONU-MPPs. Here, the transmitted optical power of the OLT is restrained by the CO due to the CO controlling both wireless components and optical components in the envisioned FiWi access networks. In the downlink direction, the four OSUs used for data communication and the one OSU applied to the PoF technology are firstly converted into an optical signal in an arrayed waveguide grating router (AWGR), then transmitted over the multimode fiber-enabled optical distribution networks between the OLT and the ONU-MPP. Usually, the primary remote node (RN) contains a splitting ratio of 1:4, and the secondary RN can adopt a typical splitting ratio of 1:64. 

## 4. Problem Formulation of Energy Consumption Minimization

In this section, the energy consumption of all the integrated ONU-MPPs is provided by PoF technology instead of external power supply, which is represented by high costs and operational spending. In addition, a joint bandwidth allocation and sleep scheduling scheme of integrated ONU-MPPs is introduced, which is crucial to improving energy efficiency. Finally, the energy supply required by the integrated ONU-MPPs scheduling strategy is taken into consideration via PoF-enabled harvested power technology.

### 4.1. Joint Bandwidth Allocation and Power-Saving Method

**Figure 2:**
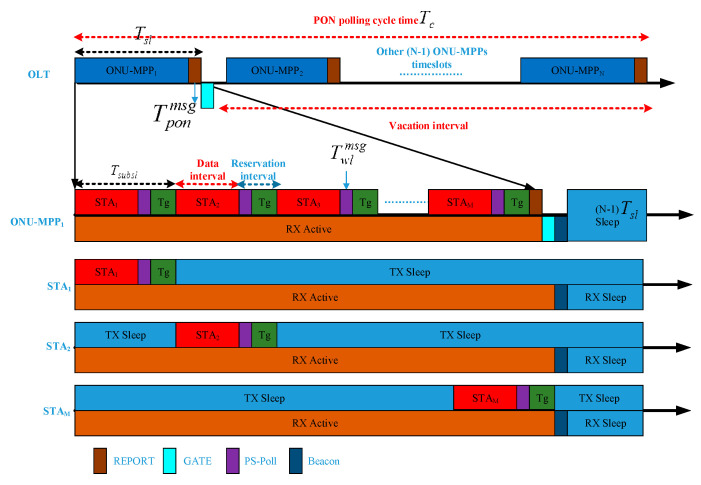
Power-saving illustration of the FiWi access network.

Unlike the fact that some remote radio heads (RRHs), consisting of an ONU module, battery module, and antenna module, can switch from the active state into sleeping mode only if the residual energy of the battery module in the RRH is below a certain level [25], without taking into consideration the real-time traffic requirements of the aware ONU-MPP sleep scheduling strategy. In this paper, however, each integrated ONU-MPP allocates a subslot to its associated wireless STAs in order to achieve a bandwidth allocation and energy-saving mode scheduling as shown in Figure 2. Here, in order to notably achieve energy conservation, it is clear from Figure 2 that in a PON polling cycle time $T_{c}$, an OLT can provide polling service for N ONU-MPPs. Any integrated ONU-MPP only operates in a certain timeslot $T_{sl}$, and then sleeps in other timeslots $\left(N-1\right)T_{sl}$, and its timeline consists of a data interval, reservation interval, and vacation interval in the uplink, receiving data internally in the downlink. Besides, an ONU-MMP associates M STAs, where an STA transmits data to the associated ONU-MPP in a small timeslot, and then enters into a sleep state while receiving data from an ONU-MPP in a certain timeslot $T_{sl}$. In other words, the duration length of the active state and sleep state is defined as $T_{a}^{i}$, $T_{a}^{i}=T_{sl}$, where i is the index of the integrated ONU-MPP, i.e., i∈[1,2,…,N] and $T_{s}^{i}$, $T_{s}^{i}=\left(N-1\right)T_{sl}$, respectively. The set of integrated ONU-MPP branches and that of each ONU-MPP accommodates STAs are denoted by $O=\left\{o_{1},\ldots ,o_{i},\ldots ,o_{N}\right\}$ and $S=\left\{s_{i,1},\ldots ,s_{i,j},\ldots ,s_{i,M}\right\}$, j∈[1,2,…,M], respectively. In addition, the polling cycle time $T_{c}$ is denoted by the summation of both the ONU-MPPs’ allocated timeslot $T_{sl}$ and wake-up overhead time $T_{onu\text{-}mpp}^{oh}$, which ensures all the ONU-MPP components can enter the sleep phase in every polling cycle time. Meanwhile, the achieved bandwidth $B_{i}$ for the converged ONU-MPP $o_{i}$ is represented by the total bandwidth B provided by the OLT and the total number of ONU-MPP accommodated STAs, i.e., $B_{i}=\frac{B}{N}$.

In each uplink timeslot $T_{sl}$ allocated by the OLT, the STA $s_{i,j}$ successively transmits an uplink data interval, a *PS-poll frame*, and a guard frame, which is used to mitigate the interference between the two adjacent STAs, whereas always keeping the TX sleep state in the residual timeslot of the allocated timeslot $T_{sl}$. Therefore, each STA must possess an active phase of $T_{subsl}$ and a sleep phase of $T_{sl}-T_{subsl}$. Meanwhile, all the STAs still receive the downlink data traffic from the associated ONU-MPP in the allocated timeslot $T_{sl}$. More specifically, due to the PoF technology-enabled power supply, the energy consumption of the envisioned FiWi access network is calculated for an ONU-MPP component as follows:

(1) $$E_{c}=p_{onu\text{-}mpp}^{a}\left(T_{sl}+T_{onu\text{-}mpp}^{oh}\right)+p_{onu\text{-}mpp}^{s}\left(T_{c}-T_{sl}-T_{onu\text{-}mpp}^{oh}\right)$$

where $p_{onu\text{-}mpp}^{a}$ and $p_{onu\text{-}mpp}^{s}$ denote the power consumed by the active and sleep mode of each converged ONU-MPP, respectively. For the sake of simplification, we can assume that the energy consumption in the wakeup process is the same as that in the active phase. However, the total energy consumption of a traditional ONU-MPP in a polling cycle time can be calculated as

(2) $$E=T_{c}\cdot p_{onu-mpp}^{a}$$

In other words, the accommodated ONU-MPP is always in an active state. When the energy-saving scheme is taken into consideration, the energy efficiency in the considered FiWi access networks is verified as follows:

(3) $$\eta =\frac{E-E_{c}}{E}$$

In this regard, after substituting the polling cycle time and Equation (2) into Equation (3), Equation (3) can be further rewritten as $\eta =\frac{N-1}{N}\cdot \frac{p_{onu\text{-}mpp}^{a}-p_{onu\text{-}mpp}^{S}}{p_{onu\text{-}mpp}^{a}}$. It is worth mentioning that the energy efficiency η is denoted as a function of both the total number of converged ONU-MPPs and the power consumption in a different state mode for the sleep scheduling paradigm and the harvested power strategy. Give the potential advantages of the PoF technology, the effect of the transmitted optical power on the energy harvest is remarkable due to the optical power that can be transformed into electrical power in the ONU module.

### 4.2. PoF Technology-Enabled Power Supply

In our envisioned FiWi broadband access network, the crucial CO contains multiple OLTs represented by the set $L\in \left\{l_{1},l_{2},\ldots ,l_{k},\ldots ,l_{L}\right\}$. Similarly, four OSUs are used for data communication, but the remaining one is PoF technology via a dedicated optical wavelength and accommodates four integrated ONU-MPP branches. In the TWDM-PON utilizing PoF technology, the transmitted optical power originating from the OSUs in OLT is susceptible to optical fiber power loss, which is the result of the fiber attenuation factor of both the feeder fiber and distribution fiber, segregation ratio of the passive splitter in the RN, and photoelectric conversion efficiency at the photodiode (PD) component in the ONU module. Therefore, the electrical power of the ONU-MPP $o_{i}$ receiving from the OLT $l_{k}$ is attenuated, and further can be expressed as follows:

(4) $$P_{l_{k},o_{i}}^{rx}=\frac{P_{l_{k}}^{tx}\cdot \Gamma \left(d_{l_{k},o_{i}}\right)\cdot \zeta }{N}$$

where $P_{l_{k}}^{tx}$ and $P_{l_{k},o_{i}}^{rx}$ are the transmitted optical power of the OLT $l_{k}$ and the received power of the electrical signal for the ONU-MPP $o_{i}$ addressed to the OLT $l_{k}$, respectively. Furthermore, ζ, $d_{l_{k},o_{i}}$, and N are defined as the photoelectric conversion efficiency of the PD, the link transmission distance between the OLT $l_{k}$ and the ONU-MPP $o_{i}$, and the segregation ratio of the RN, respectively. Given the effect of link transmission distance and fiber attenuation factor on the optical fiber power loss factor $\Gamma \left(d_{l_{k},o_{i}}\right)$, $\Gamma \left(d_{l_{k},o_{i}}\right)=10^{(-d_{l_{k},o_{i}})\cdot \alpha /10}$ explains the mutual relationship in detail, in which α is the fiber attenuation factor in dB/km.

The receiving component Rx of the battery module can convert the transmitting unnecessary electrical signal after the PD component into available electrical power. In addition, the battery module of the integrated ONU-MPP can always store the harvested electrical power during the sleep phase or active phase. The whole evolution process is referred to as PoF technology. Note, however, that the harvested electrical power of the battery module in the ONU-MPP $o_{i}$ both in the sleep and active mode is differentiated, and can be calculated as Equations (5) and (6), respectively.

(5) $$HP_{l_{k},o_{i}}^{s}=P_{l_{k},o_{i}}^{rx}$$

(6) $$HP_{l_{k},o_{i}}^{a}=P_{l_{k},o_{i}}^{rx}\left(1-\frac{\beta_{l_{k},o_{i}}}{N}\right)$$

where $\beta_{l_{k},o_{i}}$ is the link resource utilization between the OLT $l_{k}$ and the ONU-MPP $o_{i}$. Therefore, the harvested electrical energy of the ONU-MPP $o_{i}$ in a polling cycle time can be derived as follows:

(7) $$HE_{l_{k},o_{i}}=HP_{l_{k},o_{i}}^{a}\left(T_{sl}+T_{onu\text{-}mpp}^{oh}\right)+HP_{l_{k},o_{i}}^{s}\left(T_{c}-T_{sl}-T_{onu\text{-}mpp}^{oh}\right)$$

It can be anticipated that the effect of the transmitted optical power $P_{l_{k}}^{tx}$ on the harvested electrical energy is remarkable. In order to facilitate the energy-efficient operation of the envisioned FiWi access network, it is required along with the strict constraint, i.e., $HE_{l_{k},o_{i}}\geq E_{c}$.

### 4.3. Correlation between Multi-Hops and QoE Value

As shown in Section 4.1 and Figure 2, any integrated ONU-MPP polls the data interval $X_{u}$, the reservation interval $V_{u}$ on the incorporation of the *PS-poll frame* $T_{wl}^{msg}$, the guard interval $T_{g}$, i.e., $V_{u}=T_{wl}^{msg}+T_{g}$, as well as the vacation interval $S_{u}$ on the combination of the residual timeslot duration and multi-point control protocol (MPCP) messaging time, i.e., $S_{u}=\left(N-1\right)\cdot T_{sl}+T_{pon}^{msg}$ in the uplink scheduling direction. In view of the data–reservation–vacation-based polling system, the dependence of the polling cycle time $T_{c}$ on the non-data H2H transmission ration $1-\rho_{u}^{h2h}$ can be further expressed as follows:

(8) $$T_{c}=\frac{N\cdot \left(M\cdot V_{u}+RTT\right)}{1-\rho_{u}^{h2h}}$$

where $\rho_{u}^{h2h}$ is the uplink aggregated H2H traffic load intensity and $\rho_{u}^{h2h}\in [0,1)$, RTT is usually represented by $2\cdot T_{prop}$, which is the propagation delay from the OLT $l_{k}$ to the integrated ONU-MPP $o_{i}$. The mean end-to-end H2H packet delay crossing from the associated STA to the sustainable OLT in the CO is decomposed into the WMN frontend mean packet delay and TWDM-PON backhaul mean packet delay.

Here, for the sake of simplicity, it is clear that the converged ONU-MPP in the polling cycle time $T_{c}$ starts with the sleep phase, and then goes through the active phase and ends with the sleep phase. Each ONU-MPP designed to M associated STAs is modeled as the *M/G/1* queue paradigm with a reservation interval and vacation interval, while each MP/MAP is in the WMN frontend *M/M/1* model. In the multi-hop WMN subnetwork, all the SATs firstly access the near MAP, then relay the multiple MPs, and finally arrive at the accommodated ONU-MPP in the first come first served (FCFS) manner. Similarly to the envisioned directed connectivity graph *G(N, E)* proposed in [30], from a perspective of mesh topology, the mean packet delay stemming from the source node represented by the STA $s_{i,j}$ to the destination node in terms of the ONU-MMP $o_{i}$ can be computed as follows:

(9) $${\bar{D}}_{wmn}^{j,i}=\sum_{h=1}^{H}\left(d_{h}^{tras}+d_{h}^{prop}+d_{h}^{syn}+d_{h}^{que}\right)$$

where $d_{h}^{tras}$, $d_{h}^{prop}$, $d_{h}^{sys}$, and $d_{h}^{que}$ are the transmission delay over the point-to-point (P2P) link, propagation delay over the P2P link, synchronization delay, and queuing delay over the MAP/MP, respectively. In addition, H elaborates on the total number of hops in an effective critical path according to the delay-aware routing algorithm (DARA) [31]. In particular, when it comes to the fact that the transmission distance between the nodes is extremely adjacent in the WMN frontend, the propagation delay $d_{h}^{prop}$ from the STA $s_{i,j}$ to the ONU-MPP $o_{i}$ can be neglected. The effect of transmission delay $d_{h}^{tras}$, synchronization delay $d_{h}^{sys}$, and queuing delay $d_{h}^{que}$ on the mean packet delay is critical, and accordingly can be computed as $\frac{1}{\mu_{h}\cdot C_{h}}$, $\frac{1}{2\mu_{h}\cdot C_{h}}$, and $\frac{\rho_{h}}{\mu_{h}\cdot C_{h}-\lambda_{h}}$, where $C_{h}$ is link capacity, $\rho_{h}$ denotes link load intensity, $\lambda_{h}$ indicates packet arrival rate, and $\mu_{h}$ represents service rate. Therefore, Equation (9) can be further rewritten as Equation (10).

(10) $${\bar{D}}^{j,i}{}_{wmn}=\sum_{h=1}^{H}\left(\frac{1}{\mu_{h}\cdot C_{h}}+\frac{1}{2\mu_{h}\cdot C_{h}}+\frac{\rho_{h}}{\mu_{h}\cdot C_{h}-\lambda_{h}}\right)$$

On the other hand, both the frame queuing delay and frame processing delay over the converged ONU-MPP $o_{i}$ also should be taken into consideration, except for the propagation delay between the ONU-MPP $o_{i}$ and OLT $l_{k}$ in the TWDM-PON optical backhaul with a gated service. It is worth mentioning that both $T_{a}^{i}\neq 0$ and $T_{s}^{i}\neq 0$ can provide a power-efficient service for the end-users. We can also assume that the US aggregate traffic arrival rate in the OLT $l_{k}$ is $\lambda_{u}$, and therefore $\frac{\lambda_{u}}{N}$ is the US traffic arrival rate of the integrated ONU-MPP $o_{i}$ and its associated STAs. Given the paramount importance of the delay analysis, the first- and second-order moments of the US data frame service time is denoted by $\bar{X_{u}}$ and $\bar{X_{u}^{2}}$, while the first- and second-order moments of the US data frame reservation time are $\bar{V_{u}}$ and $\bar{V_{u}^{2}}$, respectively. In addition, the US data frames are assumed to arrive at the converged ONU-MPP $o_{i}$ buffer by right of the Poisson Process and $\rho_{u}^{h2h}=\lambda_{u}\cdot \bar{X_{u}}$ can be expected to be achieved. The US end-to-end delay ${\bar{D}}_{pon}$ in the backhaul TWDM-PON is decomposed into the average frame queue delay $\bar{Q_{u}}$, average frame processing service delay $\bar{X_{u}}$, and frame propagation delay $T_{prop}$, i.e., ${\bar{D}}_{pon}^{i,k}=\bar{Q_{u}}+\bar{X_{u}}+T_{prop}$. Furthermore, the queue delay $\bar{Q_{u}}$ in the envisioned energy-saving FiWi access network can be written as

(11) $$\bar{Q_{u}}=\frac{N}{N-\rho_{u}^{h2h}}\left(\bar{R_{u}}+\bar{Y_{u}}\right)$$

where $\bar{R_{u}}$ and $\bar{Y_{u}}$ define the mean frame residual delay and the mean frame reservation and vacation delay, respectively. Reference [14] serves as a basis for the mean frame queuing delay $\bar{Q_{u}}$, which can be rewritten as

(12) $$\begin{array}{l} \bar{Q_{u}}=\frac{\rho_{u}^{h2h}\cdot \bar{X_{u}^{2}}}{2(N-\rho_{u}^{h2h})\cdot \bar{X_{u}}}+\frac{(1-\rho_{u}^{h2h})V_{u}}{2(N-\rho_{u}^{h2h})}+\frac{{\left(N-1\right)}^{2}\cdot (MV_{u}+RTT)}{2(N-\rho_{u}^{h2h})\cdot (1-\rho_{u}^{h2h})}\\ +\frac{\left[MV_{u}(N-\rho_{u}^{h2h})+(N-1)RTT]\cdot (MN+M-\rho_{u}^{h2h}\right)}{M(N-\rho_{u}^{h2h})\cdot (1-\rho_{u}^{h2h})}-\frac{\left(M^{2}+M-2\right)V_{u}}{2M(N-\rho_{u}^{h2h})} \end{array}$$

In the envisioned joint bandwidth allocation and power-saving scheme, the mean US frame end-to-end delay of the optical backhaul subnetwork is computed by adding up the average frame queue delay, average frame processing service delay, and frame propagation delay.

(13) $$\begin{array}{l} {\bar{D}}_{pon}^{i,k}=\frac{\rho_{u}^{h2h}\cdot \bar{X_{u}^{2}}}{2(N-\rho_{u}^{h2h})\cdot \bar{X_{u}}}+\frac{(1-\rho_{u}^{h2h})V_{u}}{2(N-\rho_{u}^{h2h})}+\frac{{\left(N-1\right)}^{2}\cdot (MV_{u}+RTT)}{2(N-\rho_{u}^{h2h})\cdot (1-\rho_{u}^{h2h})}\\ +\frac{\left[MV_{u}(N-\rho_{u}^{h2h})+(N-1)RTT]\cdot (MN+M-\rho_{u}^{h2h}\right)}{M(N-\rho_{u}^{h2h})\cdot (1-\rho_{u}^{h2h})}-\frac{\left(M^{2}+M-2\right)V_{u}}{2M(N-\rho_{u}^{h2h})}+\bar{X_{u}}+T_{prop} \end{array}$$

Finally, after combining Equations (10) and (13), the end-to-end US frame delay in the envisioned FiWi broadband access network can be derived as follows:

(14) $${\bar{D}}_{u}^{e2e}={\bar{D}}_{wmn}^{j,i}+{\bar{D}}_{pon}^{i,k}$$

From Equation (14), the effect of the total number of STAs M, the total number of ONU-MPP N, the total number of the hop H, the aggregate frame US arrival rate $\lambda_{u}$, the reservation interval $V_{u}$, and propagation delay $T_{prop}$ on the end-to-end frame delay is obvious.

To the best of our knowledge, the network QoS in terms of the packet loss ratio increases along with the increased communication distance between the RRHs and end-users in the emerging CRAN, and the QoE value accordingly decreases [25]. Similarly, with the increasing multi-hops between the ONU-MPP and end-users, the mean end-to-end frame delay rises gradually, and the QoE value decreases gradually with the increase in the mean end-to-end frame delay. From the perspective of the network, according to the average data obtained in the test, a corresponding mean opinion score (MOS) value under different QoS conditions is first obtained, and then the numerical calculation software MATLAB is used to fit the results according to the specific function model, and, finally, the expression between the QoE and network delay is obtained. According to the investigation result achieved in Reference [32], the dependence of the QoE value Q on the mean end-to-end frame delay in the FiWi access network ${\bar{D}}_{u}^{e2e}$ can be given by

(15) $$Q=2.03e^{-2.94{\bar{D}}_{{}_{u}}^{e2e}}+2.39.$$

## 5. Numerical Analysis and Discussion

In this section, the performance evaluation of the PoF-enabled energy harvest over the envisioned FiWi access network, which considers not only the QoE-guaranteed network operation as a function of the end-to-end traffic delay but also the minimization of energy consumption via joint bandwidth allocation and a sleep scheduling scheme, is presented and discussed based on the valid parameter settings.

### 5.1. Parameter Settings

As shown in Table 1, the parameter setting is presented before the experimental simulation. From the perspective of a QoE-guaranteed network operation alongside energy consumption minimization, providing the polling cycle time of the TWDM-PON is vitally challenging, having a relation with the total number of converged ONU-MPP in each branch, the number of STAs accommodated by each ONU-MPP, the reservation interval, the round-trip time between the OLT and ONU-MPP, and the US aggregated traffic load intensity, which are denoted by N, M, $V_{u}$, RTT, and $\rho_{u}^{h2h}$, respectively. For the sake of simplicity, each integrated ONU-MPP can accommodate M STAs in our simulation area. On the other hand, the harvested electrical power in the ONU-MPP is designed to guarantee a minimum energy consumption in order to make the broadband access network operate efficiently without an external power supply. Therefore, the transmitted optical power of the OLT $l_{k}$, i.e., $P_{l_{k}}^{tx}$ depends on both $\rho_{u}^{h2h}$ and N. In addition, the communication distance $d_{l_{k},o_{i}}$ and fiber attenuation factor α correcting to the optical fiber power loss is separately defined as 100 km and 3 dB/km due to the multimode fiber being deployed in the optical backhaul network. For a network reach of 100 km, the RTT can be assumed to equal 1 ms, and the propagation delay $T_{prop}$ is equal to 0.5 ms. Then, both the photoelectric conversion efficiency ξ and link resource utilization $\beta_{l_{k},o_{i}}$ related to the conversation efficiency equaled 0.6 and 1%, respectively. Given the paramount importance of energy efficiency in both the envisioned FiWi access networks and the traditional PON access network, the power consumption of the ONU-MPP and ONU in the active and sleep state was set as 5552 mW and 5052 mW as well as 758 mW and 750 mW, respectively.

### 5.2. Performance Evaluation

**Figure 3:**
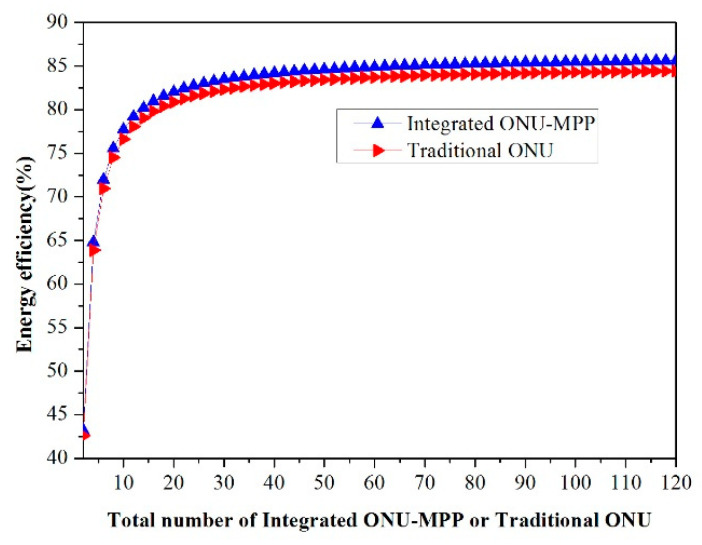
Energy efficiency η vs. the total number of integrated ONU-MPPs or traditional ONU N.

Figure 3 illustrates the energy efficiency vs. the total number of emerging integrated ONU-MPP or single traditional ONU for different access network architectures. Clearly, from Figure 3, the energy efficiency firstly increases along with the increasing total number of integrated ONU-MPP or tradition ONU until the number reaches 30, and then rises gradually. In addition, the energy efficiency of the integrated ONU-MPP is superior to that of the traditional access network paradigm. According to a further simplified form of Equation (3), we know that the energy efficiency of the proposed power-saving method entirely depends on the total number of ONU-MPP and the power consumption in different operating states. We can also assume that the maximum energy efficiency of both access networks is 86.3% and 85.2% due to the total N in Equation (3) coming close to positive infinity. More specifically, when N equals 30, the energy efficiency of the two paradigms is 83.5% and 82.3%; when N reaches 60, their energy efficiencies are approximately 86.3% and 85.2%, respectively.

**Figure 4:**
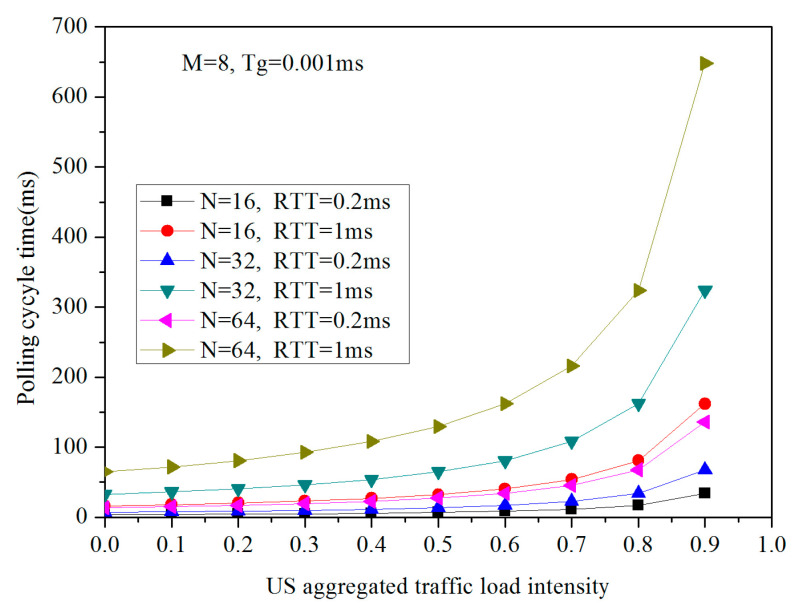
$T_{c}$ vs. $\rho_{u}^{h2h}$ for variable values of N and RTT.

**Figure 5:**
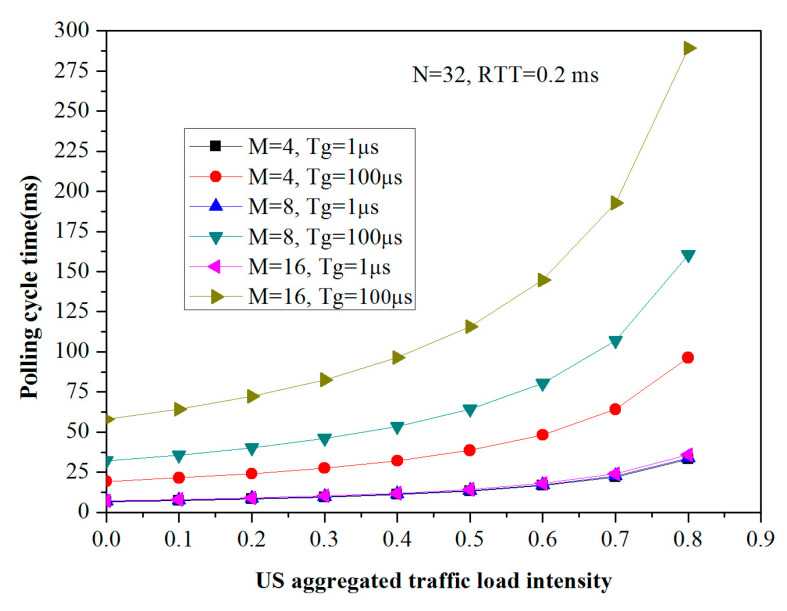
$T_{c}$ vs. $\rho_{u}^{h2h}$ for variables of M and $T_{g}$.

The polling cycle time is calculated for dynamic resource allocation in the envisioned FiWi access networks, in which the dependence of $T_{c}$ on N, M, $T_{wl}^{msg}$, $T_{g}$, RTT, and $\rho_{u}^{h2h}$ is obvious, and Equation (8) provides the in-depth technical guidelines in our simulation. Figure 4 and Figure 5 describe the polling cycle time $T_{c}$ as a function of N, RTT, M, and $T_{g}$. Given the fact that $T_{wl}^{msg}$ is constant due to the size of the PS-poll frame, the Beacon frame is set to 64 bytes. Here, for the sake of tractability, both N and RTT are variable in the presence of M and $T_{g}$ is set to 8 and 0.001 ms, respectively. We can observe from Figure 4 that the polling cycle time $T_{c}$ rises gradually with the increasing US aggregated traffic load intensity $\rho_{u}^{h2h}$ for any given configuration. After exceeding the load intensity of 0.5, the polling cycle time $T_{c}$ sharply increases accordingly. More specifically, the polling cycle time of RTT equaling 0.2 ms is significantly lower than that of the RTT setting of 1 ms in the same amount of ONU-MPPs. In other words, the larger number of the ONU-MPP, or the longer optical network reach, the larger the polling cycle time. It is import to note, however, that $T_{c}$ could be less than 10 ms when the N is 16, the RTT is set to 0.2 ms, and the load intensity $\rho_{u}^{h2h}$ is less than 0.7. When the frame delay requested by the user equipment is tolerant, it is not necessary to set the value of $T_{c}$ lower. On the other hand, when N and RTT is regularly set to 32 and 0.2 ms, respectively, M and $T_{g}$ is critical to $T_{c}$, as depicted in Figure 5. With the increase in the US aggregated traffic load intensity, the polling cycle time rises considerably; especially, $T_{c}$ could be very short, i.e., has less than a 10 m response time, even if the network architecture has 16 STAs accommodated in each ONU-MPP, the guard time is set to 1, and is operating at a utilization factor of 0.6. However, the $T_{c}$ could be adjusted by extending the reservation interval $V_{u}$ according to the end-user tolerant frame delay. In addition, as the $T_{g}$ increase varying from 1 μs to 100 μs, $T_{c}$ almost increases doubly. In a word, we can notice from Figure 4 and Figure 5 that both N and RTT have a more significant influence on $T_{c}$ than both M and $T_{g}$.

**Figure 6:**
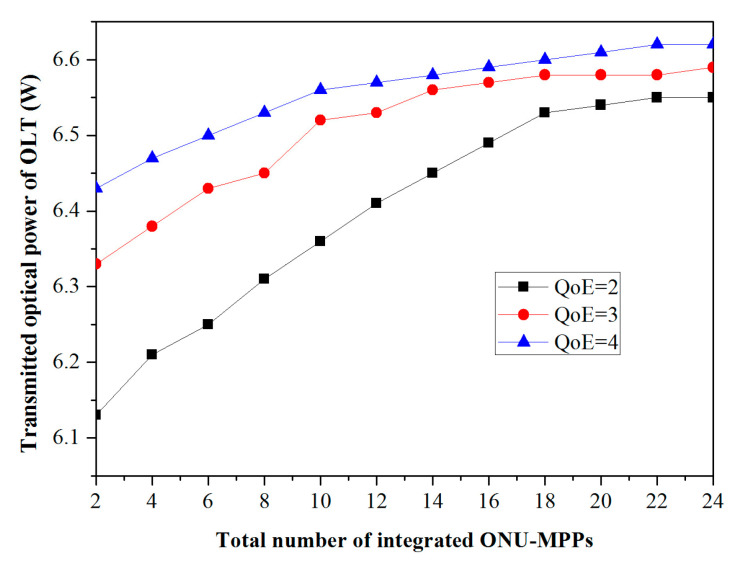
Different QoE values.

In Figure 6, we clearly observe that the average transmitted optical power of the OLT vs. the total number of integrated ONU-MPPs for different QoE values is described in greater detail based on the above Equations (1) and (7). As the total number of integrated ONU-MPPs increases, the averaged transmission optical power of the OLT increases under the same QoE level. This is because the larger value of N will lead to a longer interval of $T_{c}$, as depicted in Figure 4, which then contributes to a higher energy consumption of the ONU-MPP, and finally being conducive to a higher transmitted optical power, applying the PoF technology to supply the energy of the ONU-MPP. In view of the QoE-guaranteed network operation, the transmitted optical power is not restricted to N but to the QoE level. Similarly, as can be seen from Figure 6, the transmitted optical power increases as the QoE level increases even in the same number of integrated ONU-MPPs. Notably, the larger the value of the QoE level, the shorter the end-to-end frame delay that the US scheduling scheme can achieve from Equation (7), so that the accommodated ONU-MPPs is still active for an extended period of time, and can consume more energy via increasing the power of emitting light. In turn, the transmitted optical power is significantly decreased under the lower QoE level. More specifically, when the values of N exceed 20, the transmitted optical power tends to be stable for the requested QoE level; the result is the same as the description from Figure 2. Meanwhile, the explicit values of the QoE values for the 2nd, 3rd, and 4th levels are defined as 6.55 W, 6.59 W, and 6.62 W, respectively. 

**Figure 7:**
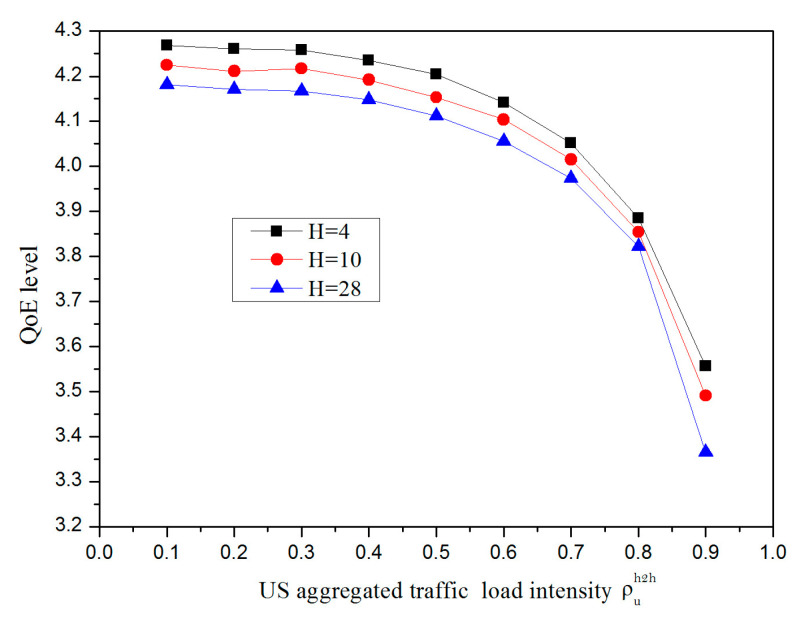
Impact of $\rho_{u}^{h2h}$ on the QoE for different hops.

It is challenging to ensure that our proposed verification method is created with a QoE value as a function with an end-to-end US frame delay in mind. It is worth mentioning that the wireless frontend hops H and uplink load intensity $\rho_{u}^{h2h}$ codetermines the end-to-end US frame delay. Here, we can assume that the total number of integrated ONU-MPPs, N, is 32; the total number of STAs provided by each ONU-MPP M can set as 8; the guard interval $T_{g}$ is denoted as 1μs; and the second-order moment of frame service time $\bar{X_{u}^{2}}$ equals 21.44 μs^2^. As can be seen from Figure 7, when the US-aggregated traffic load intensity increases, the QoE value decreases gradually; this experimental find was calculated based on Equations (14) and (15). This is because the larger value of the US load intensity makes the end-to-end delay increase, and further decreases the QoE level. Besides, as the number of wireless hops H increases in the same $\rho_{u}^{h2h}$ situation, the QoE value decreases sharply. For example, when the $\rho_{u}^{h2h}$ varies from 0.1 to 0.7 by taking into consideration the WMN frontend consisting of a total number hops of 28, the end-to-end frame delay is less than 10 ms when computed via Equation (15), and in turn a QoE value higher than the 4th level under Equation (5). Importantly, the network operators provide mobile users with an acceptable QoE level by ensuring the number of multiple hops subjected to the threshold.

## 6. Conclusions

In this paper, an envisioned FiWi broadband access network, integrating the WMN frontend subnetwork and TWDM-PON optical backhaul and adapting PoF technology, is proposed. To evaluate network performance, we took the joint bandwidth allocation and ONU-MPP sleep scheduling strategy, PoF-enabled electrical power harvest, and a correlation between multi-hops and the QoE value into consideration. Experimental results show that a business-driven ONU-MPP sleep paradigm outperforms the traditional power-saving scheme. The polling cycle time as a function of US-aggregated traffic load intensity was discussed in detail. In addition, the transmitted optical power can adjust dynamically according to both the QoE level and the total number of integrated ONU-MPPs. Finally, the network operators can achieve an energy-saving target by adjusting the suitable number of hops in the WMN.

## Figures and Tables

**Table 1:** Parameters settings used in the envisioned FiWi access network.

Parameter	Description	Value
N	Number of integrated ONU-MPPs	16, 32, 64
M	Number of STAs associated with ONU-MPP	4–20
H	Number of multi-hops	4–30
$\rho_{u}^{h2h}$	US aggregated traffic load	(0, 1)
$T_{wl}^{msg}$	WMN message time	0.512 μs
$T_{g}$	Guard time between the consecution STA’s subslot	1–100 μs
$T_{c}$	Polling cycle time	ms
$\bar{X_{u}}$, $\bar{X_{u}^{2}}$	First- and second-order moments of frame service time	5.09 μs, 21.44 μs^2^
$T_{prop}$	Propagation delay between OLT and ONU-MPP	0.1–0.5 ms
$p_{onu\text{-}mpp}^{a}$, $p_{onu}^{a}$	Power consumption in active state	5552 mW, 505 mW
$p_{onu\text{-}mpp}^{s}$ $p_{onu}^{s}$	Power consumption in sleep state	758 mW, 750 mW
$P_{l_{k}}^{tx}$	Transmitted optical power of the OLT $l_{k}$	W
$d_{l_{k},o_{i}}$	Communication distance between OLT $l_{k}$ and ONU-MPP $o_{i}$	100 km
ξ	Photoelectric conversion efficiency	0.6
α	Optical fiber attenuation factor	3 dB/km
$\beta_{l_{k},o_{i}}$	Link resource utilization between OLT $l_{k}$ and ONU-MPP $o_{i}$	1%

## References

[1] Vereecken W., Heddeghem W.V., Deruyck M., Puype B., Lannoo B., Joseph W., Colle D., Martens L., Demeester P. (2011). Power Consumption in Telecommunication Networks: Overview and Reduction Strategies. IEEE Commun. Mag..

[2] Fu S., Wen H., Wu J., Wu B. (2017). Cross-Networks Energy Efficiency Tradeoff: From Wired Networks to Wireless Networks. IEEE Access.

[3] Marcus M. (2015). 5G and “IMT for 2020 and beyond”. IEEE Wirel. Commun..

[4] Maier M., Levesque M., Ivanescu L. (2012). NG-PONs 1&2 and Beyond: The Dawn of the Uber-FiWi Network. IEEE Netw..

[5] Aurzada F., Levesque M., Maier M., Reisslein M. (2014). FiWi Access Networks Based on Next-Generation PON and Gigabit-Class WLAN Technologies: A Capacity and Delay Analysis. IEEE/ACM Trans. Netw..

[6] Sarkar S., Yen H.H., Dixit S., Mukherjee B. (2009). Hybrid Wireless Optical Broadband Access Network (WOBAN): Network Planning using Lagrangean Relaxation. IEEE/ACM Trans. Netw..

[7] Huang M., Chen Y., Peng P., Wang H., Chang G. (2020). A Full Field-of-View Self-Steering Beamformer for 5G mm-Wave Fiber-Wireless Mobile Fronthaul. J. Lightwave Technol..

[8] Dat P.T., Kanno A., Yamamoto N., Kawanishi T. (2019). Seamless Convergence of Fiber and Wireless Systems for 5G and Beyond Networks. J. Lightwave Technol..

[9] Van D.P., Rimal B.P., Chen J., Monti P., Wosinska L., Maier M. (2016). Power-Saving Methods for Internet of Things over Converged Fiber-Wireless Access Networks. IEEE Commun. Mag..

[10] Maier M., Levesque M. (2014). Dependable Fiber-Wireless (FiWi) Access Networks and Their Role in a Sustainable Third Industrial Revolution Economy. IEEE Trans. Reliab..

[11] Sarigiannidis A.G., Iloridou M., Nicopolitidis P., Papadimitriou G., Pavlidou F.-N., Sarigiannidis P.G., Louta M.D., Vitsas V., Antonios S., Maria I. (2015). Architectures and Bandwidth Allocation Schemes for Hybrid Wireless-Optical Networks. IEEE Commun. Surv. Tut..

[12] Hou W., Ning Z., Guo L. (2018). Green Survivable Collaborative Edge Computing in Smart Cities. IEEE Trans. Ind. Inform..

[13] Van D.P., Rimal B.P., Andreev S., Tirronen T., Maier M. (2016). Machine-to-Machine Communications Over FiWi Enhanced LTE Networks: A Power-Saving Framework and End-to-End Performance. J. Lightwave Technol..

[14] Raavi S., Andrade M.D., Fiandra R., Tornatore M. Energy-efficient Design and Equipment Placement for Wireless Optical Broadband Access Networks. Proceedings of the 2012 IEEE Online Conference on Green Communications (GreenCom).

[15] Van D.P., Rimal B.P., Maier M., Valcarenghi L. (2016). ECO-FiWi: An Energy Conservation Scheme for Integrated Fiber-Wireless Access Networks. IEEE Trans. Wirel. Commun..

[16] Chowdhury P., Tornatore M., Sarkar S., Mukherjee B. (2010). Building a Green Wireless Optical Broadband Access Network (WOBAN). J. Lightwave Technol..

[17] Coimbra J., Schtz G., Correia N. Network Game based Routing for Energy Efficient Fibre-Wireless Access Networks. Proceedings of the IEEE International Conference on Communications.

[18] Kantarci B., Naas N., Mouftah H. Energy-efficient DBA and QoS in FiWi networks constrained to metro-access convergence. Proceedings of the 14th International Conference on Transparent Optical Networks.

[19] Schutz G., Correia N. (2012). Design of QoS-aware Energy-efficient Fiber Wireless Access Networks. IEEE/OSA J. Opt. Commun. Netw..

[20] Gong X., Hou W., Guo L., Zhang L. (2012). Dynamic Energy-saving Algorithm in Green Hybrid Wireless Optical Broadband Access Network. Optik.

[21] Liu J., Guo H., Fadlullah Z.M., Kato N. (2016). Energy Consumption Minimization for FiWi Enhanced LTE-A HetNets with UE Connection Constraint. IEEE Commun. Mag..

[22] Guo H., Liu J., Fadlullah Z.M., Kato N. (2018). On Minimizing Energy Consumption in FiWi Enhanced LTE-A HetNets. IEEE Trans. Emerg. Top. Comput..

[23] Ahmed M., Ahmad I., Habibi D. (2015). Service Class Resource Management for Green Wireless-Optical Broadband Access Networks (WOBAN). J. Lightwave Technol..

[24] Miyanabe K., Rodrigues T.G., Lee Y., Nishiyama H., Kato N. (2019). An Internet of Things Traffic-Based Power Saving Scheme in Cloud-Radio Access Network. IEEE Internet Things J..

[25] Suto K., Miyanabe K., Nishiyama H., Kato N., Ujikawa H., Suzuki K.-I. (2015). QoE-Guaranteed and Power-Efficient Network Operation for Cloud Radio Access Network with Power Over Fiber. IEEE Trans. Comput. Soc. Syst..

[26] Miyanabe K., Suto K., Fadlullah Z.M., Nishiyama H., Kato N., Ujikawa H., Suzuki K.-I. (2015). A cloud radio access network with power over fiber toward 5G networks: QoE-guaranteed design and operation. IEEE Wirel. Commun..

[27] Togashi K., Nishiyama H., Kato N., Ujikawa H., Suzuki K.-I., Yoshimoto N. (2013). Cross Layer Analysis on ONU Energy Consumption in Smart FiWi Networks. IEEE Wirel. Commun. Lett..

[28] Han P., Guo L., Liu Y., Hou J., Han X. (2016). Joint Wireless and Optical Power States Scheduling for Green Multi-Radio Fiber-Wireless Access Network. J. Lightwave. Technol..

[29] Nishiyama H., Togashi K., Kawamoto Y., Kato N. (2015). A Cooperative ONU Sleep Method for Reducing Latency and Energy Consumption of STA in Smart-FiWi Networks. IEEE Trans. Parallel Distrib. Syst..

[30] Fadlullah Z.M., Nishiyama H., Kato N., Ujikawa H., Suzuki K.-I., Yoshimoto N. (2013). Smart FiWi Networks: Challenges and Solutions for QoS and Green Communications. IEEE Intell. Syst..

[31] Sarkar S., Yen H.-H., Dixit S., Mukherjee B. (2008). A Novel Delay-aware Routing Algorithm (DARA) for a Hybrid Wireless-Optical Broadband Access Network (WOBAN). IEEE Netw..

[32] Fiedler M., Hossfeld T., Tran-Gia P. (2010). A Generic Quantitative Relationship between Quality of Experience and Quality of Service. IEEE Netw..

